# LILRA3 is related to monocyte-derived dendritic cell maturation and activation 

**DOI:** 10.22038/ijbms.2020.50714.11555

**Published:** 2021-02

**Authors:** Xinyu Wu, Qi Cheng, Huawei Jiang, Meiju Zhou, Xiaochan Chen, Huaxiang Wu, Jing Xue, Yan Du

**Affiliations:** 1 Department of Rheumatology, The Second Affiliated Hospital, Zhejiang University School of Medicine, 88 Jiefang Road, Hangzhou, 310009, China; 2 Department of Hematology (Cancer Institute, Key Laboratory of Cancer Prevention and Intervention, China National Ministry of Education, Key Laboratory of Molecular Biology in Medical Sciences, Zhejiang Province, China), The Second Affiliated Hospital, Zhejiang University School of Medicine, 88 Jiefang Road, Hangzhou, 310009, China

**Keywords:** Cell differentiation, Dendritic cell, JAK/STAT1, LILRA3, Monocyte, MAPK p38

## Abstract

**Objective(s):**

Previously we reported functional leukocyte immunoglobulin-like receptor A3 (*LILRA3*) leads to susceptibility and sub-phenotypes of several autoimmune diseases. LILRA3 levels in blood serum and CD14^+^ monocytes enhanced in systemic lupus erythematosus and resulted in disease severity. However, the mechanism of LILRA3 in the pathogenesis of autoimmunity remains elusive. This study aims to explore the potential impact of LILRA3 on the differentiation, maturation, and function of monocyte-derived DCs (MoDCs).

**Materials and Methods:**

The human monocytic cell line (THP-1) was cultured to derive MoDCs in vitro. We performed plasmid transfection to examine the impact of LILRA3 on monocyte differentiation. Surface markers on MoDCs were measured using FACS. To assess the function of mature MoDCs, IL-12p70, IFN-γ and IL-4 levels were detected after the mixed leucocyte response by enzyme-linked immunosorbent assay. Western blot assay was employed in this study to determine the signaling pathways in MoDCs activation.

**Results:**

LILRA3 promotes MoDCs maturation, our results showed significant up-regulation of CD40, CD80, CD86, CD209, and HLA-DR and increased production of pro-inflammatory cytokine IL-12. LILRA3-treated MoDCs exhibited a robust proliferation of allogeneic CD4^+^ T cells and induced naïve CD4^+ ^T cell polarization into the Th1 phenotype. Furthermore, the preceding activation of MoDCs maturation and LILRA3 function might be attributed to p38 MAPK and STAT1 signaling pathway’s aberrant activation.

**Conclusion:**

This is the first study to report that LILRA3 played a critical role in promoting MoDCs maturation and directing MoDCs to modulate Th1 cell differentiation, which may have a role in the pathogenesis of autoimmune diseases.

## Introduction

Immune responses are the result of interaction between non-specific immune response and specific immune response. Dendritic cells (DCs), have vital functions in immune surveillance and prevent infection and tumors and are abundantly present omnipotent antigen-presenting cells (APCs) (1). DCs have an important part in host immunity by triggering endogenous immune reactions to pathogens, stimulating memory T cells, effectively priming naive T cells, and encouraging B cell stimulation. DCs are also needed to sustain steady-state immune homeostasis through continuously introducing tissue-derived self-antigens to CD8^+^ and CD4^+^ T cells in the absence of inflammatory signals, contributing to self-antigen tolerance. DCs can influence the initiation of tolerance and immunity in a variety of ways, as well as during DC growth, the characteristics of DC subsets, and the degree of DC maturation.

According to the recent simplified nomenclature, DCs comprise four classes, monocyte-derived DC cells (MoDC), pDC, cDC1, and cDC2 (2). Based on this grouping, steady-state DCs can be classified into 3 subsets according to their origin, surface markers and functions: plasma cell-like DCs (pDCs) that can produce high levels of type 1 interferon (IFN), and two conventional DC (cDC) subpopulation, cross-presenting cDC1 expressing CD8 or CD103 in mice and cDC2 expressing CD11b + and effectively stimulating the proliferation of CD4 + T cells. Besides these steady-state DC subsets, the genetic signatures of human DC subsets found in tissues clearly indicate that in addition to the previously mentioned DCs originating from devoted precursors (pre-DCs), monocyte-derived DCs (MoDCs) still occur in human tissues (3). Due to the low amount of DCs in human peripheral blood, MoDCs are commonly utilized as an *in vitro* model to research the function and formation of DCs. MoDCs are derived from peripheral blood monocytes utilizing IL-4 and GM-CSF. These are similar to natural sources of blood DCs in the capacity to modulate costimulatory molecules in reaction to maturation signals and to pose entrapment antigens to T lymphocytes (4). 

Immunoglobulin-like transcripts (ILTs) also called leukocyte immunoglobulin-like receptors (LILRs) belong to 13 strongly homologous immunomodulatory proteins clustered of human chromosome 19 in the vicinity of 19q13.4 (5, 6). LILRA3 belongs to the LILR family that is produced by monocytes and macrophages in the form of a soluble molecule. A 6.7-kb deletion in the LILRA3 gene has been reported that causes a null allele and an absence of function (6, 7). The incidence of the 6.7-kb deletion varies among different populations, with a large rise in Northeast Asians (0.56–0.84) relative to Africans (0.10) and Europeans (0.17) (8). The nonfunctional *LILRA3 *homozygous LILRA3 deletion was correlated with Sjogren’s syndrome (SS) and multiple sclerosis (MS) in Caucasians (9-11). Conversely, in our early research, we observed that in the Chinese, non-deletion of LILRA3 (namely functional LILRA3) leads to subphenotypes and sensitivity of rheumatoid arthritis, SS, and systemic lupus erythematosus (12, 13).

LILRA3 is mainly produced by monocytes, Low *et al.* reported that recombinant LILRA3 could not only induce the expression of IL-6 and IL-1α but also alter the costimulatory molecules and MHC expression in B cells and monocytes (14). However, to the best of our knowledge, LILRA3’s part in the stimulation of human DCs phenotypic and characteristics are elusive. This study aims to advance the knowledge of LILRA3-induced MoDC maturation, function, and associated signaling pathways.

## Materials and Methods

***Isolation of T cells***

Venous blood samples were obtained from healthy individuals. Human PBMCs were isolated using a Ficoll method based on the density difference between PBMCs and other components in the blood. CD4^+ ^T cells were isolated from PBMCs via the CD4^+^ T Cell Isolation Kit, LS separation column, and MidiMACS^TM^ separator. Next, sorted cells were evaluated by flow cytometric analysis with purity in excess of 95%. The freshly isolated T cells were adopted to detect mixed leucocyte responses (MLRs).

***Lentiviral transduction***

The lentivirus vector for LILRA3 over-expression was established by pGC-LV-GV287-GFP vector recombining with LILRA3 (NM_006865.4) gene, defined as pLVX-LILRA3. Whereas, the lentivirus vector for LILRA3 knockdown was obtained by cloning small hairpin RNAs (shRNA) utilizing a self-inactivating lentivirus vector of CMV-driven GFP reporter and a U6 promoter (Gene Chem, Shanghai, China), defined as LILRA3 shRNA. Three target sequences for LILRA3 were designed and we choose the best for further study. According to the instructions, in a 24-well plate, when the cell reached sixty percent confluence, they were transfected lentivirus vector at a 10 MOI. The next morning, the medium was changed with a fresh medium to keep the cells healthy. After 48 hr, the efficiency of transfection was verified by Western blotting. 

***Cell culture and generation of LILRA3-affected MoDCs from THP-1 cells***

The differentiation of THP-1 cells was induced according to a previous protocol (15). Briefly, THP-1 cells were grown in RPMI 1640 with 10% fetal bovine serum, 1% penicillin and streptomycin, and 2 mmol/l L-glutamine in an incubator containing 5% CO_2_ at 37 °C. Various recombinant cytokines of human origin were used to stimulate the cells, including recombinant human GM-CSF (50 ng/ml), IL-4 (20 ng/ml), and TNF-α (20 ng/ml) (peprotech systems). 

To establish MoDCs, IL-4 and GM-CSF were supplemented in the THP-1 cells at the indicated concentrations for 1 week. To generate LILRA3-affected MoDCs, pLVX-LILRA3 or LILRA3-shRNA was utilized to transfect the THP-1 cells, and 48 hr after transfection , GM-CSF/IL-4 were used to stimulate the cells for 1 week. The MoDCs derived from THP-1 transfected with pLVX-NC or shLILRA3-NC served as controls. Mature and active MoDCs were generated from THP-1 cells that were treated with GM-CSF/IL-4 for 1 week (16) and additionally culture for 2 more days with TNF-α (17). Cells were fed every three days by changing the medium and complementing new fresh medium with the above-mentioned full cytokines doses.

***Morphological examination***

To investigate the impact of LILRA3 on monocyte differentiation, the morphology of MoDCs informed by transfecting with pLVX-LILRA3, shLILRA3, pLVX-NC, or shLILRA3-NC was evaluated every other day through an Olympus inverted microscope. 

***Flow cytometric analysis***

MoDCs were detected using the following antibodies, FITC anti-human CD1a (Biolegend), PE anti-human CD209, CD86, CD80, CD40, and HLA-DR (Biolegend).

***Mixed leucocyte responses***

Active and mature MoDCs established as mentioned earlier were harvested and γ-irradiated (2.25 Gy/min) after incubation with 3 x 10^3^ allogeneic CD4^+^ T cells/well, at ratios of MoDCs to T cells of 1:10, 1:50, and 1:100 (18). After 3 days, CCK8 was added (10 μl/well) and incubated the plates for 4 hr. Then, absorbance at 450 nm was determined through a microplate reader.

***Enzyme-linked immunosorbent assay***

MoDCs were established as mentioned earlier. IL-12p70 secreted by MoDCs differentiated from THP-1 transfected with pLVX-LILRA3, shLILRA3, pLVX-NC, or shLILRA3-NC was measured with an ELISA kit (R&D system).

To examine the effect of LILRA3-over-expressed MoDCs on T lymphocyte polarization, T lymphocytes were cultured with γ-irradiated LILRA3-over-expressed MoDCs at a 1:10 MoDCs to T lymphocytes ratio for 7 days. 20 ng/ml of TNF-α was supplemented and the cells were grown for 2 more days. IFN-γ and IL-4 concentration were measured from the collected suspension through human IFN-γ and IL-4 ELISA kits (R&D). 

***Western blotting***

MoDCs in a six-well plate were collected and lysed in a radioimmunoprecipitation (RIPA) lysis buffer with 1% 100 mmol PMSF (phenylmethylsulfonyl fluoride) and then for 30 min it was incubated on ice (frequent shaking the plate). The lysates were centrifuged at 4 °C, 12,000 x g for half an hour to remove cell fragments and nucleic acids. Half of the supernatants were collected to detect protein concentration by the BCA method, and the other supernatants were diluted with the loading buffer and boiled for 15 min. After cooling to ambient temperature, it was then used for WB analysis.

Next, we prepared the SDS-PAGE. 10% separation glue and 5% concentration glue were prepared to separate equal amounts of protein at 100-120 V constant voltage electrophoresis. The glue after electrophoresis was transferred onto polyvinylidene fluoride (PVDF) membranes (Immobilon-P; Millipore, Billerica, MA, USA). The membranes were blocked for 2 hr and then incubated with numerous diluted (5% BSA and TBST) primary antibodies, at 4 °C with careful vibrating till morning. The next morning the membrane was washed with TBST on a decolorizing table concentrator at room temperature 3 times. The PVDF membrane was incubated with secondary antibody (horseradish peroxidase-conjugated, diluted with TBS to 1:1000) at room temperature for 60 min. After washing with TBST, the blots were established by an increased chemiluminescence detection kit (Pierce Biotechnology, Inc., Rockford, IL, USA), and GAPDH served as control. 

***Statistical analysis***

We performed the statistical analysis using SPSS 25.0 statistical software package (SPSS, Inc., Chicago, IL, USA) and GraphPad Prism 8.0 (GraphPad Software, Inc., San Diego, CA, USA). All quantitative data are expressed as mean±SD. *P*-value<0.05 was considered as significant difference.

## Results

***The lentiviral transfection efficiency and its effect on the LILRA3 expression in THP-1 cells***

**Figure 1:**
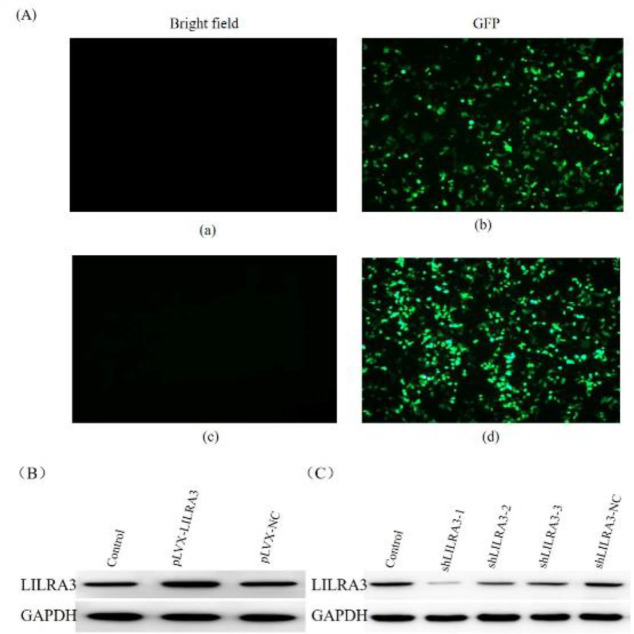
The lentiviral transfection efficiency and its effect on LILRA3 expression in THP-1 cells. (A) The expression of LILRA3 with GFP at 48 hr after transfection. (a) and (c) bright field, (b) LILRA3-over-expression, (d) LILRA3 knockdown; (B) and (C) Western blotting confirmed LILRA3 expression after lentiviral transfection

Due to the difficulty of transfecting primary monocytes, in this study, we chose THP-1 cells, a human leukemic monocyte-macrophage cell line, as the model for monocyte-macrophage differentiation. We first explored the impact of pLVX-LILRA3 and shLILRA3 on LILRA3 expression in THP-1 cells. To this end, we transfected THP-1 cells with a lentiviral construct encoding LILRA3 enhancer (pLVX-LILRA3), LILRA3 inhibitor (shLILRA3), or control virus (control group). GFP signals were observed using a fluorescence inverted microscope 48 hr after transfection (Figure 1A). Western blotting analysis of the transfected THP-1 cells confirmed that LILRA3 over-expression or knockdown could effectively enhance or inhibit the target LILRA3 compared with the control group and the un-transfected cells (Figures 1 B and C). In the knockdown experiments, three target sequences for LILRA3 were designed, and the best one (shLILRA3-1) was used for further study.

***Effect of LILRA3 on the morphological changes in the differentiation of monocytes into dendritic cells***

**Figure 2:**
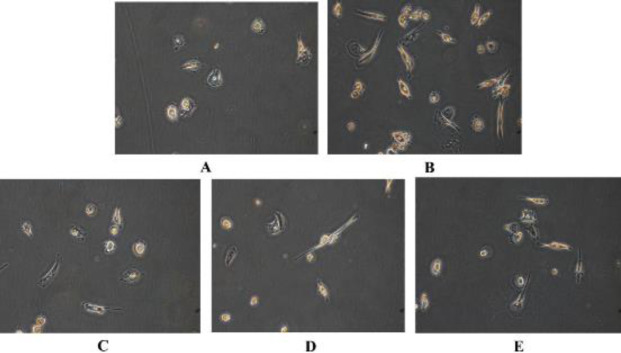
Morphological comparison of monocyte-derived DCs (MoDCs) transfected with pLVX-LILRA3, shLILRA3, pLVX-NC, shLILRA3-NC, or control cells by inverted microscope. Cell morphology changes were observed to evaluate the effects of LILRA3 on MoDCs differentiation upon treatment with GM-CSF/IL-4 for 1 week, followed by stimulation with TNF- α for another 2 days. (A) THP-1 cells without transfection treated with GM-CSF/IL-4/TNF-α; (B) The human monocytic cell line (THP-1) cells were transfected with pLVX-LILRA3, 48 hr later cells were further treated with GM-CSF/IL-4/TNF-α; (C) THP-1 cells were transfected with shLILRA3, 48 hr later cells were further treated with GM-CSF/IL-4/TNF-α; (D) THP-1 cells were transfected with pLVX-NC, 48 hr later cells were further treated with GM-CSF/IL-4/TNF-α; (E) THP-1 cells were transfected with shLILRA3-NC, 48 hr later cells were further treated with GM-CSF/IL-4/TNF-α. All above cells were observed with an inverted microscope (x 200)

We observed THP-1 cells are capable to differentiate into mature MoDCs after 1 week of supplementation with GM-CSF/IL-4, then stimulating with TNF-α for 48 hr (Figure 2A). We constructed pLVX-LILRA3 (LILRA3-over-expression) or pLVX-NC (control group) in THP-1 cells using lentiviral transfection and determined whether they can be differentiated into MoDCs. We found that GM-CSF/IL-4 /TNF-α induced MoDCs can differentiate in pLVX-LILRA3 and pLVX-NC transfected THP-1 cells. Compared with pLVX-NC transfected THP-1 cells (Figure 2D), the number of MoDCs differentiated from pLVX-LILRA3 transfected THP-1 cells was significantly increased (Figure 2B). Based on our observations, we constructed LILRA3-knockdown THP-1 cells using lentiviral transfection to examine LILRA3’s effects on MoDCs differentiation. The number of MoDCs differentiated from shLILRA3 transfected THP-1 cells (Figure 2C) was significantly decreased compared with shLILRA3-NC transfected THP-1 cells (Figure 2E). 

***LILRA3 up-regulates surface molecules expressed on MoDCs***

**Figure 3:**
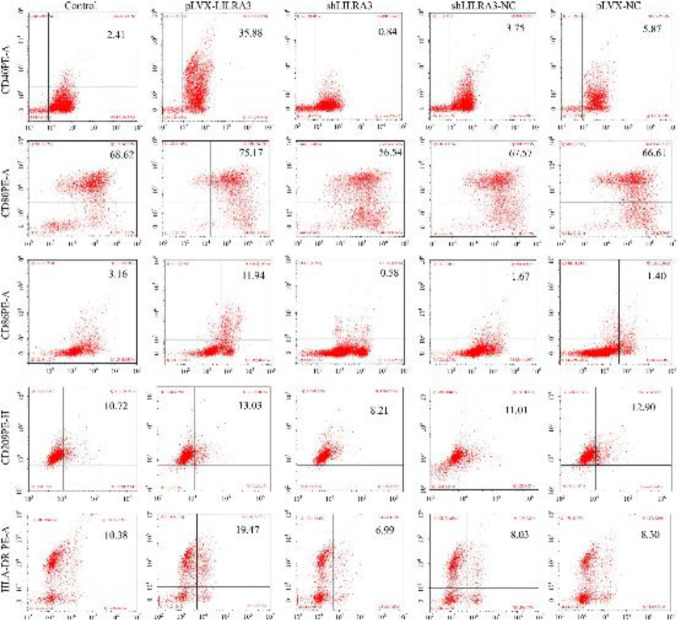
Comparison of the phenotypic profiles of monocyte-derived DCs (MoDCs) differentiated from THP-1 cells that experienced different treatment. MoDCs primed with pLVX-LILRA3, shLILRA3, pLVX-NC, or shLILRA3-NC transfection were incubated with fluorochrome-conjugated monoclonal antibodies (mAbs), and the antigens of CD40, CD80, CD86, CD209, HLA-DR, and CD1a on the surface of those cells were analyzed by flow cytometry. All experiments were performed in triplicates. MoDCs differentiated from LILRA3-over-expression THP-1 cells expressed higher levels of CD40, CD80, CD86, CD209, and HLA-DR than those MoDCs differentiated from THP-1 cells without transfection. MoDCs differentiated from LILRA3-knockdown THP-1 cells expressed lower levels of CD40, CD80, CD86, CD209, and HLA-DR than did those MoDCs differentiated from THP-1 cells with no transfection

After treatment with GM-CSF/IL-4/TNF-α, THP-1 cells, LILRA3-over-expression THP-1 cells, LILRA3-knock down THP-1 cells and THP-1-control-vector cells were collected. FACS was employed to assess the expression of DC related surface markers. We found that MoDCs differentiated from LILRA3-over-expression THP-1 cells expressed CD40, CD80, CD86, CD209, and HLA-DR in higher amounts than MoDCs differentiated from THP-1-control-vector or LILRA3-knockdown THP-1 cells (*P*<0.05 or *P*<0.01, Figure 3, Table 1 and Table 2).

***LILRA3 enhances the MoDCs potential to present antigens to allogeneic T lymphocytes***

**Figure 4:**
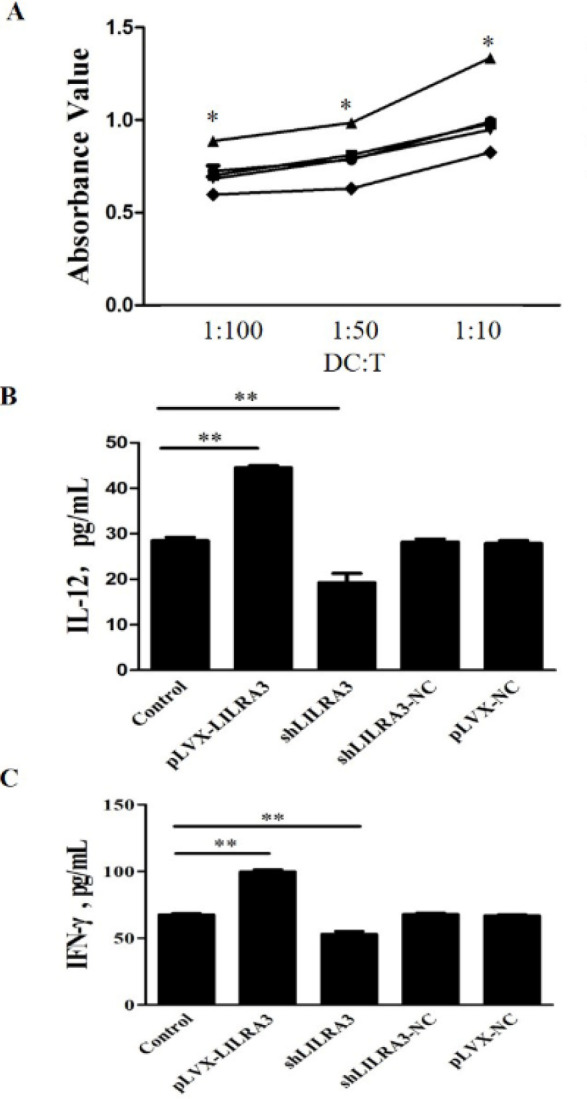
Functional analysis monocyte-derived DCs (MoDCs) differentiated from THP-1 cells that experienced different treatment. A) Effect of LILRA3 on modulating the ability of MoDCs to present antigens to T cells was examined by allogeneic mixed leukocyte responses (MLRs). B) Effect of LILRA3 on IL-12 production by MoDCs. C) Analysis of cytokine-production in T cells primed with co-stimulation by MoDCs (* *P*<0.05, ** *P*<0.01)

We performed an allogeneic MLR assay to further investigate the impact of LILRA3 on regulating the potential of MoDCs to activate T cell proliferation. Mature MoDCs differentiated from pLVX-LILRA3, pLVX-NC, shLILRA3, and shLILRA3-NC transfected THP-1 cells were cultured with allogeneic CD4^+^ T cells at various ratios of MoDC to T cells. When the ratio of MoDC to T cells increases (1:100, 1:50, and 1:10), the potential to stimulate T cell proliferation is also enhanced (Figure 4A). Furthermore, at the different ratios of MoDC to T cells, the degree of T cell proliferation caused by MoDCs differentiated from pLVX-LILRA3 transfected THP-1 cells was statistically higher than those induced by MoDCs differentiated from pLVX-NC cells or shLILRA3 transfected THP-1 cells (*P*<0.05). In summary, these findings indicate that MoDCs differentiated from LILRA3-over-expressed THP-1 cells had a higher potential to induce T-cell proliferation compared with controls (Figure 4A).

***LILRA3 up-regulates IL-12 secretion by activated MoDCs***

To examine the outcome of LILRA3 on IL-12 produced by activated MoDCs, GM-CSF/IL-4 was used to activate the THP-1 cells transfected with pLVX-LILRA3, pLVX-NC, shLILRA3, or shLILRA3-NC for 1 week and treated for another 48 hr with TNF-α to generate activated and mature MoDCs. Our results releveled that activated and mature LILRA3-over-expressed MoDCs manufactured more IL-12 than MoDCs primed with pLVX-NC or shLILRA3 transfection (Figure 4B; *P*<0.01).

***TH1 polarization by LILRA3-primed MoDCs***

Next, we investigate whether LILRA3 induced the capability of MoDCs to assist T helper cell differentiation, activated and mature MoDCs were γ-irradiated then incubated with T cells for six days. The ELISA results showed that T cells activated with LILRA3-over-expression mature MoDCs produced more IFN-γ than those stimulated with MoDCs primed with pLVX-NC or shLILRA3 transfection (*P*<0.01, Figure 4C). However, there are no differences of the production of IL-4 among the above three groups (data were not shown).

***LILRA3 up-regulates intracellular protein level of MAPK p-p38 and pSTAT1***

**Figure 5:**
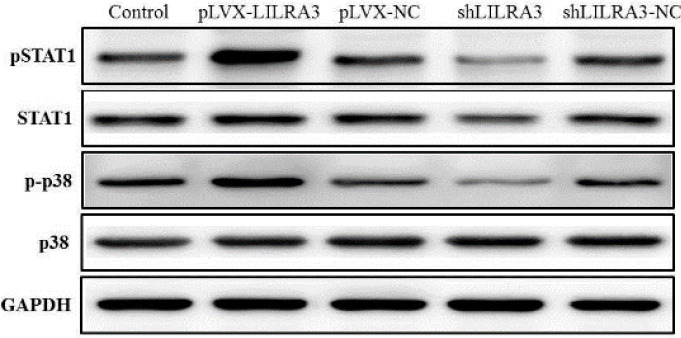
LILRA3 activated the signaling pathways controlling monocytes differentiated into MoDCs. THP-1cells transfected with pLVX-LILRA3, pLVX-NC, shLILRA3, or shLILRA3-NC were stimulated with GM-CSF/IL-4 for 1 week and further treated with TNF-α for 2 days to generate mature and activated DCLCs. Then the cells were lysed for assessment of the phosphorylation of p38 and STAT1 by Western blotting

Finally, we evaluate the effect of LILRA3 on MoDCs mature signal pathway, THP-1 cells transfected with pLVX-LILRA3, pLVX-NC, shLILRA3, or shLILRA3-NC were activated with GM-CSF/IL-4 for one week and next treated with TNF-α for 48 hr to generate mature and activated MoDCs. Activated MoDCs were collected and used to extract protein. Western Blotting findings revealed that LILRA3 enhances the differentiation and maturation of MoDCs and might depend on the activation of the signaling pathways of MAPKp38 and JAK/STAT1 (Figure 5).

**Table 1:** Comparison results in the MoDCs surface marker expression level (mean±SD, n=5, percentage)

Surface marker	Control	pLVX-LILRA3	shLILRA3	shLILRA3-NC	pLVX-NC	^a^ *P*	^b^ *P*
CD40	2.7±0.8	40.4±0.6	0.8±0.3	3.0±0.8	3.7±1.7	< 0.01	< 0.05
CD80	58.0±8.1	77.9±4.8	28.5±15.9	54.2±11.7	53.8±12.3	< 0.05	< 0.05
CD86	3.9±1.2	11.9±2.0	0.7±0.2	1.7±0.2	1.5±0.3	< 0.05	< 0.05
CD209	10.1±1.2	14.5±2.1	7.6±0.5	10.6±0.4	11.5±0.8	< 0.01	< 0.01
HLA-DR	10.7±0.6	19.6±2.1	7.6±0.4	8.8±0.9	8.9±1.1	< 0.01	< 0.01

^a^P: Control vs pLVX-LILRA3;^ b^P: Control vs shLILRA3

**Table 2:** Comparison results in the MoDCs surface marker expression level (mean±SD, n=5, mean fluorescent intensity, MFI)

Surface marker	Control	pLVX-LILRA3	shLILRA3	shLILRA3-NC	pLVX-NC	^a^ *P*	^a^ *P*
CD40	567.4±78.3	8095±740	292.4±41.4	1055±93.4	643±35.9	< 0.05	< 0.05
CD80	7668±493.6	11097±1538	6427±339.8	7904±391.4	8328±759.5	< 0.01	< 0.01
CD86	885.6±86.8	4502±751.6	620±135.8	841±109.4	791±69.4	< 0.01	< 0.01
CD209	1361±161.5	1743±198.1	1075±87.7	1279±187.4	1217±75.3	< 0.05	< 0.05
HLA-DR	1407±250.6	3378±363.5	1026±188.3	1034±149.8	1107±135.1	< 0.01	< 0.05

^a^P: Control vs pLVX-LILRA3; ^b^P: Control vs shLILRA3

## Discussion

Previously, we first reported that functional LILRA3 instead of non-functional LILRA3 was correlated with SLE in the Chinese peoples. The significant increase of disease activity in SLE patients with homozygous functional LILRA3, and the notable higher level of LILRA3 transcripts in LILRA3 carriers, supported the functional role of LILRA3 in the regulation of SLE susceptibility and disease activity (12). Moreover, in our recent study, we observed that LILRA3 levels in blood serum and CD14^+^ monocytes enhanced in SLE, which were strongly linked with the incidence and seriousness of the disease. Up-regulation of the expression of LILRA3 can act as a biomarker of SLE severity and activity (19). The pathogenesis underlying this genetic collation of functional LILRA3 remains unclear. 

Although the mechanism for undermining immune tolerance in SLE is still not fully established, DCs are assumed to play a crucial part in the initiation, amplification and perpetuation of the disease. Monocytes can differentiate into DCs under both steady-state and inflammatory state *in vivo* (20). MoDCs are crucial for SLE onset and progression. *In vitro* studies using human MoDCs and murine bone marrow-derived DCs (BMDCs) explore the cDCs roles in SLE. Apoptotic cells provoked MoDCs to endure maturing and produce a significant amount of IL-6, a crucial cytokine for Th17 polarization (21). In line, apoptotic cell-activated BMDCs presented the increased ability to persuade Th17 response (22). 

LILR molecules are extensively present on myeloid and lymphoid cells and might significantly modulate non-specific and specific immune responses (23). LILRA3 is mainly expressed by monocytes. Nevertheless, the part of LILRA3 in monocyte differentiation is elusive. We found that the number of mature MoDCs differentiated from pLVX-LILRA3 transfected THP-1 cells was higher than pLVX-NC or shLILRA3 transfected THP-1 cells (Figure 2). Compared with pLVX-NC and shLILRA3 transfection group, pLVX-LILRA3 transfected THP-1 cells in LILRA3 over-expressed, adopted DC morphology earlier, and showed higher similarity to DCs, indicating that LILRA3 endorsed the morphological changes in monocytes into DCs differentiation. 

The results of cell surface antigen detection show that compared with control MoDCs, LILRA3-over-expressed MoDCs showed a surface markers pattern, such as higher expression of HLR-DR, CD40, CD86, CD209, and CD80 (Figure 3, Table 1, and Table 2). Moreover, activated LILRA3-over-expressed MoDCs produce stronger MLR and induce more IL-12 than control cells (Figure 4). Our finding suggests that LILRA3 has an essential part in monocytes differentiated into mature DCs. 

We further found that LILRA3-over-expressed MoDCs stimulated higher levels of IFN-γ but not IL-4 generation by T cells, which means that LILRA3 might initiate DCs to modulate Th1 differentiation and favor the capability of DCs to tilt the immune response of Th1 rather than Th2 maturation.

Dendritic cell maturation requires various signaling pathways, like the mitogen-activated protein kinase (MAPK) pathway, JAK/STAT1 pathway, and the PI3K/AKT signaling pathway(24-26). Next, we decided to explore the underlying intracellular mechanisms in LILRA3-induced MoDCs maturation, we explored the impact of LILRA3 on the PI3K/AKT, STAT1, and MAPKp38 pathways. Western blot analysis revealed that (Figure 5) phosphorylation of STAT1 and MAPKp38 was significantly stimulated by LILRA3. There was no phosphorylation of AKT (data not shown), indicating that LILRA3 might induce MoDCs maturation by JAK/STAT1 and MAPKp38 pathways.

## Conclusion

Our results demonstrated that LILRA3 induces monocytes differentiation into mature DCs by JAK/STAT1 and MAPKp38 signaling pathways. Therefore, targeting LILRA3 may be a novel efficient therapeutic approach for SLE patients.
